# Detection of SARS-CoV-2 RNA by a Multiplex Reverse-Transcription Loop-Mediated Isothermal Amplification Coupled with Melting Curves Analysis

**DOI:** 10.3390/ijms22115743

**Published:** 2021-05-27

**Authors:** Igor P. Oscorbin, Georgiy Yu. Shevelev, Ksenia A. Pronyaeva, Andrey A. Stepanov, Darya V. Shamovskaya, Olga V. Mishukova, Dmitrii V. Pyshnyi, Maksim L. Filipenko

**Affiliations:** Institute of Chemical Biology and Fundamental Medicine, Siberian Branch of the Russian Academy of Sciences (ICBFM SB RAS), 8 Lavrentiev Avenue, 630090 Novosibirsk, Russia; metatezis@gmail.com (G.Y.S.); ks_pronyaeva@mail.ru (K.A.P.); stepanov_aa@cnmt.ru (A.A.S.); shamovskaya@gmail.com (D.V.S.); mishukova_olga@inbox.ru (O.V.M.); pyshnyi@niboch.nsc.ru (D.V.P.); mlfilipenko@gmail.com (M.L.F.)

**Keywords:** SARS-CoV-2, coronavirus, loop-mediated isothermal amplification, LAMP, multiplex amplification, melting curve analysis

## Abstract

Loop-mediated isothermal amplification (LAMP) is a method of nucleic acid amplification that is more stable and resistant to DNA amplification inhibitors than conventional PCR. LAMP multiplexing with reverse transcription allows for the single-tube amplification of several RNA fragments, including an internal control sample, which provides the option of controlling all analytical steps. We developed a method of SARS-CoV-2 viral RNA detection based on multiplex reverse-transcription LAMP, with single-tube qualitative analysis of SARS-CoV-2 RNA and MS2 phage used as a control RNA. The multiplexing is based on the differences in characteristic melting peaks generated during the amplification process. The developed technique detects at least 20 copies of SARS-CoV-2 RNA per reaction on a background of 12,000 MS2 RNA copies. The total time of analysis does not exceed 40 min. The method validation, performed on 125 clinical samples of patients’ nasal swabs, showed a 97.6% concordance rate with the results of real-time (RT)-PCR assays. The developed multiplexed LAMP can be employed as an alternative to PCR in diagnostic practice to save personnel and equipment time.

## 1. Introduction

The SARS-CoV-2 virus, belonging to Betacoronavirus genera, caused the COVID-19 coronavirus infection pandemic, which is continuing as of May 2021. The disease has spread throughout the world, and an urgent need has emerged for the for SARS-CoV-2 diagnostic tests. Most widely used test systems are based on real-time (RT)-PCR, requiring 1.5–2 h to obtain the final results. Meanwhile, the sharply increased pressure on diagnostic laboratories and the limited number of thermocyclers has made the time of analysis one of the factors limiting the number of tests that can be performed in a single day. In other words, techniques that are faster than qPCR are necessary to increase the throughput capacity of laboratories.

During the last several decades, the explosive development of molecular biology has brought about a vast amount of different approaches for the amplification of nucleic acids. Many of these rely on the amplification of DNA at a constant temperature, also known as isothermal amplification (NASBA, RPA, LAMP, HDA, MDA, RCA, SDA and some other approaches) [1,2,3,4,5,6,7,8]. The constant temperature of the reaction avoids the use of thermocyclers, providing the ability to miniaturize the required equipment and to develop devices for point-of-care testing. Among isothermal amplification methods, the loop-mediated isothermal amplification (LAMP) technique [8] is one of the most popular. LAMP utilizes the strand displacement activity of some DNA polymerases and two or three pairs of oligonucleotide primers. The reaction product can be visualized by means of gel electrophoresis, naked-eye colorimetric detection [9], fluorescent intercalating dyes or probes [10,11,12], turbidimetry [13] or electrochemical methods [14], either in real-time mode or after the reaction. LAMP has become the basis for multiple tests for infectious agents in humans, plants and livestock animals, such as influenza and Zika viruses, tuberculosis and malaria agents [15,16,17,18]. In terms of sensitivity and specificity, LAMP is equal to PCR, while being more resistant to inhibitors and providing results two to three times faster (30–40 min vs. 1.5–2 h).

The advantages of LAMP make it a promising method for the development of SARS-CoV-2 diagnostic tests. In 2020, several LAMP-based tests were designed; some of them have been certified for clinical use [19,20,21,22,23,24]. In most works, the real-time monitoring of LAMP results was used for assay optimization and the testing of analytical performance, whereas after the optimization, results have been observed by means of naked-eye colorimetry. Meanwhile, real-time LAMP is suitable for clinical laboratories, and could increase testing throughput by reducing analysis time.

Multiplex amplification is the simultaneous amplification of two or more DNA fragments in the same tube, which enables the concurrent detection of several pathogens and cuts testing costs. Multiplexing allows for the simultaneous amplification of internal reaction controls or internal controls in DNA or RNA isolation, which allows researchers to monitor the quality of NA isolated from samples and the performance of reagents for reverse transcription and amplification. The nature of the amplification mechanism makes the multiplexing of LAMP a challenging task. Thus, DNA polymerases employed for LAMP lack 5′–3′ exonuclease activity [25], forestalling use of hydrolyzing fluorescently labeled probes. The concatemeric nature of LAMP products, consisting of repetitive amplified DNA fragments, hinders the separation of products without additional procedures. LAMP multiplexing can be performed using endonuclease digestion followed by gel-electrophoresis [26,27], modified oligonucleotides [28,29,30] or the melting of amplification products in the presence of intercalating dyes [31,32]. In the latter case, the products are differentiated based on their characteristic melting temperatures. Several papers have described multiplex LAMP for the detection SARS-CoV-2 and other viruses [33,34].

Here, we have developed a method of SARS-CoV-2 RNA detection employing multiplex reverse-transcription loop-mediated isothermal amplification. The method couples multiplex real-time RT-LAMP with melting curves analysis, allowing for the addition of MS2 phage as an internal control in all test procedures.

## 2. Results

### 2.1. Choosing and Testing LAMP Primers

We designed the multiplex RT-LAMP for the simultaneous detection of two RNA molecules, the fragment of SARS-CoV-2 RNA (GenBank ID NC_045512.2) and MS2 phage RNA (GenBank ID NC_001417). The latter served as an internal control for RNA isolation when added to the clinical samples before the isolation procedure.

As targets for the LAMP, we selected conserved regions of SARS-CoV-2 genomic RNA, encoding E and N proteins, and two regions of MS2 phage genomic RNA. The primers were designed according to the recommendations on the primerexplorer.jp website. In total, we made two primer sets for the LAMP-based detection of SARS-CoV-2 and MS2 (Table 1).

As DNA standards for the evaluation of LAMP efficiency, we constructed plasmids based on pBlueScript II SK (+) vector and 200-bp SARS-CoV-2 and MS2 genome fragments. As RNA standards, we used RNA fragments obtained by the in vitro transcription of SARS-CoV-2 plasmid standards or MS2 genomic RNA. All standards were quantified by means of droplet digital PCR using the QX200 platform (Bio-Rad, USA).

**Figure 1:**
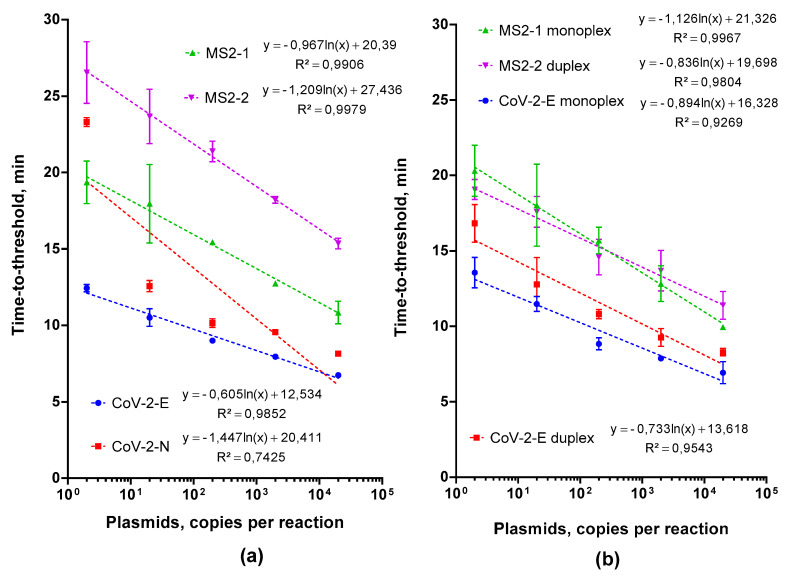
(**a**) LAMP with plasmids and different primers for SARS-CoV-2 and MS2; (**b**) LAMP with standard plasmids, monoplexes SARS-CoV-2, MS2 and duplex primer set MS2-SARS-CoV-2-E. Each primer set is marked by the color specified in the legend. Time-to-threshold (Tt) values are presented on the X-axis, and the amount of template per reaction on the Y-axis. Each run was triplicated; error bars represent one SD.

For the evaluation of real-time LAMP efficiency with the selected primers, we used serial dilutions of plasmid DNA (2 × 10^5^, two copies per reaction). The results are presented in Figure 1a. The CoV-2-E primer set showed higher amplification efficiency than the CoV-2-N set, resulting in a lower Tt values (time to reach the threshold fluorescence intensity)—in other words, a higher reaction rate. Analogous testing of LAMP primers for MS2 revealed that the set MS2-1 was more effective than MS2-2. The observed difference can be related to the complex secondary structure of DNA fragments generated with CoV-2-N and MS2-2 primers, which slows down the Gss polymerase. For further analysis, we selected CoV-2-E and MS2-1 primers.

Next, we verified the RT-LAMP efficiency, using in vitro RNA transcript standards as templates. For CoV-2-E primers, we also determined the limit of detection, i.e., the minimum amount of target RNA molecules detected by RT-LAMP. The results (Table 2) demonstrated that LAMP with both primer sets was able to detect more than 100 copies of the RNA template in the reaction. For a more precise evaluation of LAMP’s sensitivity with CoV-2-E primers, we performed 20 technical repeats with 100, 50, 20 and 10 copies of the RNA template per reaction. This experiment showed the reliable detection (20/20 technical repeats) of more than 20 molecules of SARS-CoV-2 RNA.

### 2.2. Multiplex LAMP Assay Evaluation

#### 2.2.1. Testing the Feasibility of Duplex CoV-2-E-MS2 LAMP

**Figure 2:**
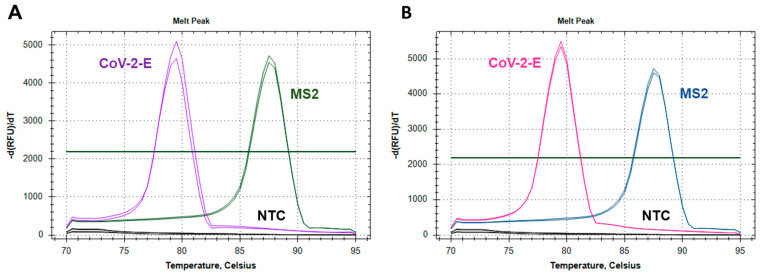
Melting curve analysis of the LAMP products. The graph represents characteristic melting peaks for LAMP products obtained with (**A**) monoplexes CoV-2-E (pink curves) and MS2 (blue curves); (**B**) duplex MS2-CoV-2-E, where green curves correspond to MS2 and purple curves to CoV-2-E.

We compared the amplification efficiencies of LAMP monoplexes CoV-2-E, MS2 and MS2-CoV-2-E duplex. For this purpose, LAMP was performed with serial dilutions of plasmid DNA CoV-2-E, MS2 and the corresponding primer sets. The duplex was tested separately with each serial dilution; CoV-2-E and MS2 primers were mixed at a 1:1 ratio. The final concentrations of primers in the duplex were equal to those in the monoplexes. Figure 1b and Figure 2 show the evaluation results. No difference in the Tt values between the monoplexes and duplex was observed for all template concentrations. Therefore, the mixing of primer sets does not hinder the either CoV-2-E or MS2 amplification. With a difference in melting temperatures of 9 °C, the melting curves of CoV-2-E and MS2 products in the duplex were clearly distinguishable.

#### 2.2.2. Adjusting Optimal Concentrations for LAMP MS2 Primers

**Figure 3:**
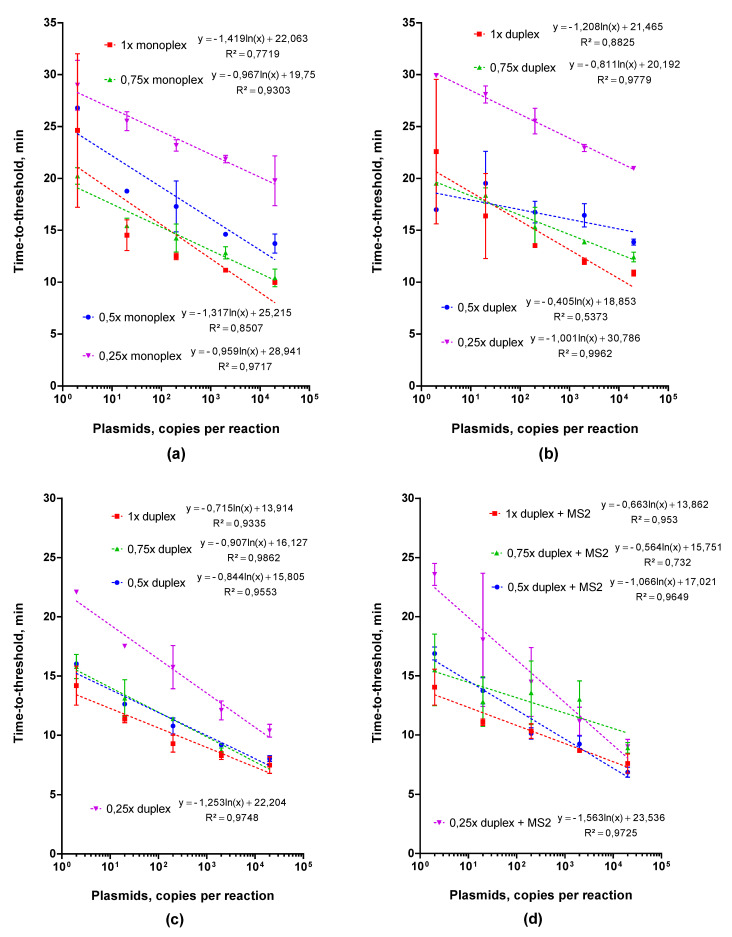
LAMP with plasmids and different concentrations of MS2 primers in (**a**) monoplex, (**b**) duplex, (**d**) duplex with MS2 RNA and (**c**) without it. Each MS2 primer concentration is marked by the color specified in the legend. Tt values are presented on the X-axis, and amount of template per reaction on the Y-axis. Each run was triplicated; error bars represent one SD.

To increase the sensitivity of the LAMP duplex assay, we varied the concentration of MS2 primers from 1× to 0.25× that of the standard, whereas the concentration CoV-2-E LAMP primers remained constant (1×). Using serial dilutions of plasmid DNA, we compared Tt values and melting curves for monoplexes of CoV-2-E and MS2 and the duplex. We also tested the amplification efficiency of the MS2-CoV-2-E duplex with the CoV-2-E plasmid on the background of the MS2 plasmid (2000 copies per reaction). The results of the comparison are presented in Figure 3. Although the concentration of MS2 primers decreased, the Tt values increased both for the monoplex and for the duplex with CoV-2-E, and the peak of the MS2 product on the melting profile flattened. For 0.25× MS2 primers, we did not observe the MS2 product peak when more than 100 copies of CoV-2-E plasmid standard were added in the reactions. Considering the Tt values in the MS2 monoplex and peak height of MS2 product in the duplex, we selected 0.5× MS2 primers for further analysis.

#### 2.2.3. Choosing an Optimal Concentration for the Control MS2 RNA

**Figure 4:**
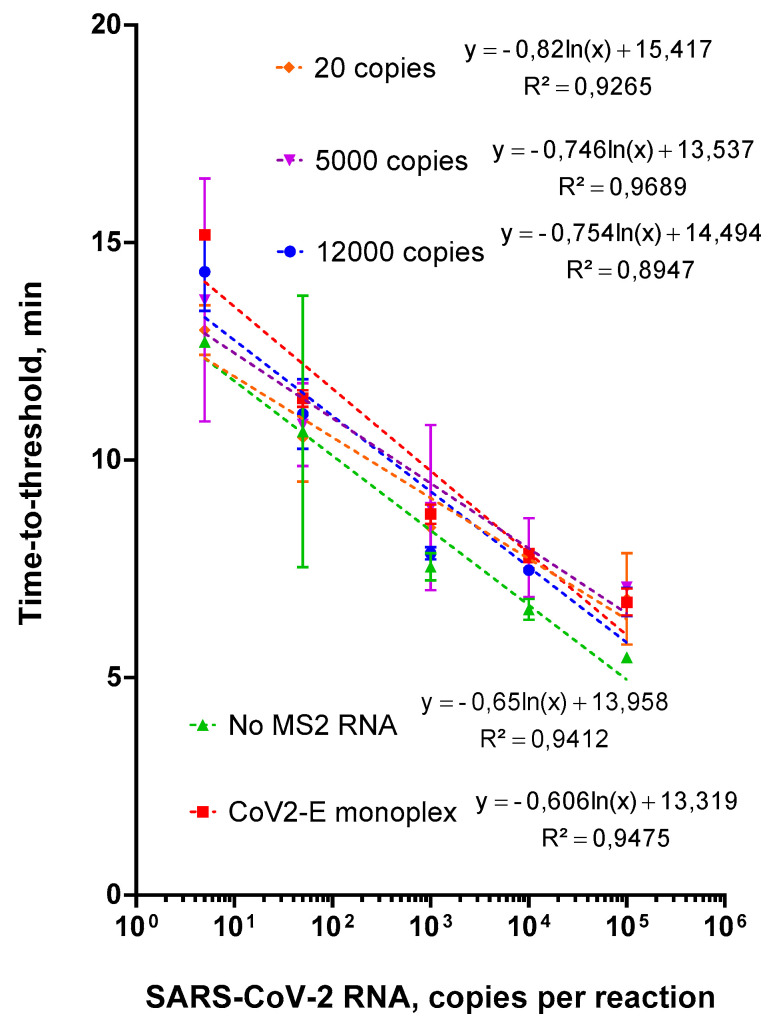
LAMP with CoV-2-E monoplex, duplex and various MS2 RNA concentrations. Each MS2 RNA concentration is marked by the color specified in the legend. Tt values are presented on the X-axis, and the amount of MS2 RNA per reaction on the Y-axis. Each run was triplicated; error bars represent one SD.

We determined an optimal concentration of the control MS2 RNA. We compared the amplification efficiency of CoV-2-E primers or the MS2-CoV-2-E duplex using serial dilutions of CoV-2-E RNA on the background of MS2 RNA (12,000, 2500 or 20 copies per reaction). The height of the corresponding peak on the melting curve served as an assessment criterion of the amplification efficiency. The results of the comparison are presented in Figure 4. The detection of more than 100 CoV-2-E RNA copies per reaction was achieved with all concentrations of MS2 RNA. However, the Tt values for 2500 and 20 MS2 RNA copies per reaction were 15 and 20 min, respectively, whereas 100 copies of CoV-2-E RNA were detected in 12 min (Figure S1). Thus, the use of fewer than 12,000 copies of MS2 RNA per reaction would only increase the total analysis time, without increasing the sensitivity of SARS-CoV-2 RNA detection. For further analysis, we used the MS2 phage as an internal control for isolation and amplification, in the amount equivalent to approximately 12,000 copies of MS2 RNA in the LAMP reaction mixture.

### 2.3. Evaluation of Duplex Assay Limit of Detection

**Figure 5:**
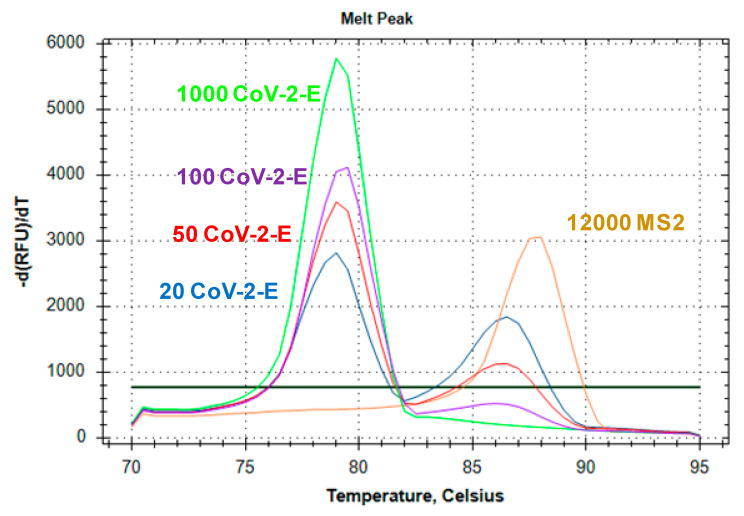
The melting curve analysis of the LAMP products: evaluation of the limit of detection for the MS2-CoV-2 duplex. The graph represents characteristic melting peaks for LAMP products obtained with the CoV-2-E monoplex and CoV-2-E RNA template (green curve), with the MS2 monoplex and MS2 RNA template (orange curve), with the MS2-CoV-2-E duplex, 12,000 copies/reaction MS2 RNA template, and with CoV-2-E RNA: 100 copies (purple curve), 50 copies (red curve) and 20 copies (blue curve).

The limit of detection (LoD) was defined as the lowest amount of SARS-CoV-2 RNA reliably detectable in a single reaction. We evaluated the LoD of the multiplex LAMP using various amount of CoV-2-E RNA, ranging from 100 to 10 copies per reaction, on the background of 12,000 copies of MS2 RNA. For each concentration, LAMP with the MS2-CoV-2-E duplex was performed in 20 technical repeats in a single run. The reaction was defined as “positive” when the peak corresponding to CoV-2-E RNA was presented on the melting curve (Figure 5). It should be noted that in the presence of more than 50 CoV-2-E RNA copies, the melting peak corresponding to MS2 RNA vanished. We marked 20 of 20 technical repeats as “positive”for 100, 50 and 20 CoV-2-E RNA molecules per reaction and 16 of 20 repeats for 10 CoV-2-E RNA molecules per reaction. The limit of detection for the multiplex LAMP was more than 20 molecules of SARS-CoV-2 RNA per reaction.

### 2.4. Testing of Clinical Samples

Multiplex MS2-CoV-2-E LAMP was validated on clinical samples of nasal swabs taken from 125 patients from CNMT ICBFM SB RAS. All patients participating in the study signed an informed consent form. Each clinical sample was spiked with 6*10^6^ MS2 phage particles before RNA purification. RNA was isolated from the samples using an AmpliSens^®^ RIBO-prep kit (Central Research Institute of Epidemiology, Moscow, Russia), then was tested by two methods: real-time RT-PCR with primers recommended by the WHO [35] and multiplex MS2-CoV-2-E LAMP (Table 1). The results of both tests were in agreement for 122 of 125 samples (Table 3). MS2 phage RNA was detected in 71.2% of samples (five SARS-CoV-2 positive and 84 negative); no MS2 peak was found in 28.8% of samples (36 SARS-CoV-2-positive). For two RT-PCR negative samples, which gave positive results according to LAMP, the Cq value of real-time RT-PCR exceeded 35. Therefore, the concordance between multiplex LAMP and real-time RT-PCR was 97.6%.

## 3. Discussion

Since the discovery of the novel SARS-CoV-2 coronavirus in December of 2019, dozens of studies have been published on LAMP-based testing for SARS-CoV-2 detection [36,37,38]. Most of these works have focused on designing cost-effective and single-tube tests suitable for point-of-care applications, such as mass home testing or testing at places of mass gathering (airports, train stations, etc.). This profound interest can be explained by the convenience of LAMP for portable testing devices, since the main advantage of LAMP is absence of the need for thermocyclers. Meanwhile, LAMP also allows for the design of tests for real-time SARS-CoV-2 detection. LAMP is two to three times faster than PCR, which saves equipment and personnel time—the most valuable resources during the current pandemic.

In the context of laboratory diagnostics, real-time detection of the pathogen provides a quantitative and semi-quantitative analysis of infectious agents. However, the option of multiplexing is equally significant, i.e., the single-tube detection of several targets saves time, consumables and samples for analysis. Multiplexing allows for the introduction of qn internal control for nucleic acid isolation and amplification, thus lowering the probability of false-negative results originating from the incorrect processing of clinical samples.

A wide spectrum of methods is available for the detection of LAMP results, from intercalating fluorescent dyes to turbidimetry. However, LAMP multiplexing remains a complicated task, requiring a solution. Here, we developed a multiplexing technique using fluorescent intercalating dye and melting curves analysis. This approach has been widely applied in PCR and has also been used for LAMP in two works [31,39]. Thus, analysis using standard real-time thermocyclers is available, without relatively expensive fluorescently labeled oligonucleotides or other specific equipment.

During the in vitro amplification of nucleic acids, the simultaneous amplification of several fragments can be hampered due to unequal reaction efficiencies for different fragments or the formation of primer dimers [40,41]. This effect has been observed for PCR but has been poorly investigated for LAMP. Considering the greater number of primers (for to six for LAMP vs. two for PCR) and their higher concentration in the reaction mixture, one should expect the same phenomenon of lowering the amplification efficiency after multiplexing to occur in LAMP. Lower efficiency can decrease the overall sensitivity of the analysis. The amplification efficiency decreased only when the simultaneous amplification of MS2 and SARS-CoV-2 fragments occurred, which consistently led to a decrease in the SARS-CoV-2 RNA detection sensitivity. We sufficiently overcame this problem by lowering the concentration of primers for the control MS2 fragment, according to the method widely used in PCR for such cases. A similar approach was applied in multiplex probe-based LAMP for the detection of SARS-CoV-2, influenza virus and human RNA as an internal control [34]. As a result, the efficiency of amplification for the MS2 phage genome fragment decreased, which improved the sensitivity of SARS-CoV-2 RNA detection using the MS2-CoV-2-E duplex to the level of CoV-2-E monoplex. MS2 fragment amplification is inhibited when more than 50 SARS-CoV-2 RNA copies are present in the reaction, making three outcomes possible: (1) MS2 fragment amplification only if the sample is SARS-CoV-2-negative, (2) SARS-CoV-2 fragment amplification only if the sample contains more than 50 SARS-CoV-2 RNA copies, and (3) amplification of both SARS-CoV-2 and MS2 fragments with two corresponding melting peaks. The latter two cases would result in SARS-CoV-2-positive samples. Therefore, MS2 amplification occurs only in the case of a low amount or absence of SARS-CoV-2 RNA, marking the importance of the proper handling of the sample before the analysis.

The characteristics of the developed duplex LAMP assay for SARS-CoV-2 RNA detection are similar to previously published analogous tests based on monoplex LAMP. The limit of detection for reported monoplex LAMP tests lies in the range of 300–10 copies of viral RNA per reaction; the concordance with PCR results was 92–95%. Therefore, the developed approach demonstrated its feasibility and can be further used to detect other nucleic acids related to other pathogens.

It is worth noting that LAMP, and the approach to multiplexing used in this study, possess some limitations. The large amount of the amplified product makes LAMP reactions a potential contamination source for lab equipment, requiring careful handling and proper waste disposal. LAMP requires more primers than PCR: six primers instead of two, and in higher concentrations. The length of BIP/FIP primers is about 40–50 nt. These two factors lead to higher requirements for the quality and quantity of synthesized oligonucleotides. LAMP also requires Bst-like DNA polymerases, which are less available compared to Taq polymerase and its analogues, which are routinely employed in PCR.

LAMP is known to be more specific than PCR due to its use of six primers instead of two. Although high specificity itself makes the test more robust and less prone to false-positive results, the detection of mutated pathogens by means of LAMP could be challenging. The SARS-CoV-2 genome is known to bear multiple mutations that could be located in the primers’ binding sites and affect the sensitivity of tests. Thus, including more target regions in the multiplex LAMP for SARS-CoV-2 detection could prevent possible false-negative results caused by mutations in primer-binding sites.

Multiplexing of LAMP is a complex task, which limits the number of pathogens for a single-tube analysis. The LAMP multiplexing method chosen in this study requires a careful selection of primers and the adjustment of melting temperatures of amplified fragments to exclude the overlapping of melting peaks. Therefore, LAMP, as well as PCR, possesses its own requirements and limitations, and the choice of testing method should be based on the careful consideration of the technique’s features and the facilities of the diagnostic lab.

We have developed a test for the detection of SARS-CoV-2 RNA based on multiplex loop-mediated isothermal amplification. Fragments of SARS-CoV-2 RNA and MS2 phage RNA were detected in a single-tube format; MS2 phage served as an internal control for RNA isolation and amplification. Multiplexing was based on the melting curves analysis of the amplification products. The limit of detection for multiplex LAMP was 20 copies of SARS-CoV-2 RNA per reaction. Testing of 125 clinical samples showed a 97.6% concordance rate with real-time RT-PCR results. The developed multiplexed LAMP can be employed as an alternative to PCR in diagnostic practice to save personnel and equipment time.

## 4. Materials and Methods

### 4.1. Clinical and Standard Samples

Clinical samples (nasal swabs) were taken from patients in the Center of New Medical Technologies ICBFM SB RAS. Plasmids with genome fragments of SARS-CoV-2 and MS2 phage, prepared using a traditional restriction-ligation cloning method, in vitro synthesized RNA fragments and MS2 phage RNA served as the control samples. For full descriptions of the cloning procedures, in vitro transcription, MS2 phage growth and MS2 phage RNA purification, see the Supplementary Materials.

### 4.2. Droplet Digital PCR

The concentrations of DNA and RNA in the obtained standards were refined by means of a digital PCR, using the QX200™ Droplet Digital™ PCR System (Bio-Rad, Hercules, CA, USA) according to the manufacturer’s instructions. The reactions were carried out in a total volume of 20 µL, containing the DNA under examination (approximately 103 copies per 20 µL), 1× ddPCR master-mix (Bio-Rad, Hercules, CA, USA), 300 nM oligonucleotide primers and probes: E_Sarb_F/R/P for CoV-2-E, N_Sarb_F/R/P for CoV-2-N [35] and MS2-5_F/R/P for MS2 phage (Table 1). For droplet generation, 20 µL of the PCR mix and 70 µL of the droplet generation oil were placed into corresponding wells of the DG8 cartridge, and the droplets were obtained in a droplet generator. Then, 40 µL of the obtained droplets were transferred to the 96-well PCR plate, foil-sealed and placed in the thermocycler. The amplification was performed using the following program: 96 °C; for 10 min, following by 45 cycles of 96 °C for 30 s, 58 °C for 60 s, with final heating for 10 min at 98 °C; the rate of plate heating was 2 °C/s for all steps. The droplets were analyzed using the droplet reader, and the obtained data were processed using the QuantaSoft package (Bio-Rad, Hercules, CA, USA).

### 4.3. Real-Time RT PCR

Real-time RT PCR was performed in a CFX 96 thermocycler (Bio-Rad, Hercules, CA, USA) in a total reaction volume of 20 µL, containing 1× PCR buffer (65 mM Tris-HCl pH 8.9, 24 mM (NH4)2SO4, 0.05% Tween 20, 2.5 mM MgCl2), 0.3 µM primers and 0.1 µM probes: E_Sarb_F/R/P for CoV-2-E and N_Sarb_F/R/P for CoV-2-N (Table 1), 1 unit of Taq-polymerase (Biosan, Novosibirsk, Russia), 100 units of M-MuLV reverse transcriptase (Biosan, Novosibirsk, Russia) and an RNA template. The amplification was performed using the following program: reverse transcription for a 10 min at 50 °C, denaturation at 95 °C for 3 min and 45 cycles with denaturation at 95 °C for 10 s, followed by annealing and elongation at 60 °C for 40 s with the registration of fluorescent signals in FAM and HEX channels.

### 4.4. Real-Time Loop-Mediated Isothermal DNA Amplification (LAMP)

The reaction mixture for LAMP (20 µL) contained 1× reaction buffer for Bst-polymerase (20 mM Tris-HCl pH 8.8, 10 mM (NH4)2SO4, 150 mM KCl, 0.1% Tween-20, 2 mM M MgSO4), 1.4 mM each of dNTP, 0.2 µM each of external primer (F3/B3), 0.6 µM loop primers (LF/BF), 1.6 µM internal primers (FIP/BIP) (Table 1), DNA or RNA template (the type and amount of the template are given below), 2 units of Gss-polymerase (EC 2.7.7.7) from Geobacillus sp. 777 [15] and intercalating dye SYTO-82 in the final concentration of 1 µM [16]. For LAMP with reverse transcription, the reaction mixture was supplied with 100 units of M-MuLV reverse transcriptase (EC 2.7.7.49, Biosan, Novosibirsk, Russia). The reactions were performed in a CFX96 thermocycler (Bio-Rad, Hercules, CA, USA). The program included the following steps: 90 cycles of primer annealing and elongation, each at 64 °C for 20 s with the registration of fluorescence signal in the HEX channel; post-amplification melting of amplification products in the range of 70 °C–95 °C. For LAMP with reverse transcription, the program was supplied with an incubation step at 50 °C for 10 min before the amplification. The results of isothermal amplification were assessed using the Tt parameter (time-to-threshold—the time interval before the intersection between an amplification curve and a threshold line). All LAMP reactions were performed in three technical replicates.

### 4.5. The Evaluation of the Limit of Detection and Clinical Sensitivity and Specificity

The limit of detection of the multiplex LAMP was assessed by varying the amount of RNA template in the reaction mixture. Multiplex LAMP was performed with 1× CoV-2-E primers and 0.5× MS2 primers under the conditions described above. The amount of CoV-2-E RNA template in the reaction mixture was 100, 50, 20 or 10 copies, on the background of 12,000 copies of MS2 phage RNA. Multiplex LAMP was performed in 20 technical repeats; CoV-2-E RNA amplification was registered as a characteristic peak on the melting curve of the amplification products. The limit of detection was defined as the concentration of CoV-2-E RNA that provided the appearance of a melting peak for CoV-2-E amplification products in all technical repeats.

Analytical specificity was evaluated in silico by checking the homology of primers with the genomes of most widespread human viral pathogens causing respiratory infections. The comparison was made using the BLAST algorithm on the NCBI website.

The clinical sensitivity and specificity of the multiplex LAMP were assessed using clinical samples (nasal swabs) taken from the patients of CNMT ICBFM RAS. RNA was isolated from clinical samples using an AmpliSens^®^ RIBO-prep kit (Central Research Institute of Epidemiology, Moscow, Russia) according to the manufacturer’s protocol. Each clinical sample was spiked with 6 × 10^6^ MS2 phage particles before RNA purification. Purified RNA was tested in the presence of SARS-CoV-2 RNA using real-time PCR and multiplex LAMP.

## Figures and Tables

**Table 1:** List of oligonucleotide primers and probes.

Name	5′-Sequence-3′
PMTL-1	CTTCGCTATTACGCCAGCT
PMTL-2	GCGGATAACAATTTCACACAG
CoR1-F2	GCCTTTGTAAGCACAAGCTG
CoR1-B2	AAGAAGGTTTTACAAGACTCACGT
CoR1-LF	CATCCTTACTGCGCTTCGAT
CoR1-LB	TAACGTACCTGTCTCTTCCGAAA
CoR1-FIP	GCAAGAAAAAGAAGTACGCTATTAACTAGAGTACGAACTTATGTACTCATTC
CoR1-BIP	CGTGGTATTCTTGCTAGTTACACTAGATATTGCAGCAGTACGCACAC
CoR2-F2	TGCAACTGAGGGAGCCTTG
CoR2-B2	TGGAGTTGAATTTCTTGAACTG
CoR2-LF	CGGCAGTCAAGCCTCTTCTC
CoR2-LB	ATTGTTAGCAGGATTGCGGGT
CoR2-FIP	GGAAGTTGTAGCACGATTGCAGATACACCAAAAGATCACATTGG
CoR2-BIP	GCTTCTACGCAGAAGGGAGCATGCGACTACGTGATGAGGAA
MS2-1-FIP	CTCCTGAGGGAATGTGGGAACCCCGGCGTGCGCGTTAT
MS2-1-BIP	GCCAGCGAGCTCTCCTCGGGCACCCGTGCTCTTTCGA
MS2-1-F3	CCGACAGCATGAAGTCCG
MS2-1-B3	AGCCCGCCCACCTTTC
MS2-1-LB	GTTAGCCACTCCGAAGTGCG
MS2-1-LF	GCTGACCGAGGGACCCC
MS2-2-LB	GTCTATACCAACGGATTTGAGCC
MS2-2-LF	GCATCCGATTCCATCTCCGAT
MS2-2-F3	TGCCTGTAAGGAGCCTGAT
MS2-2-B3	TGAGCGGATACGATCGAGAT
MS2-2-FIP	GCCAGACGCTGGTTGATCGATTAAGGGGTCGGTGCTTTCA
MS2-2-BIP	GGTTCGCTTGCGACGATAGACTTCTGGTGGGAGAAAACTCCA
N_Sarb_F	CACATTGGCACCCGCAATC
N_Sarb_R	GAGGAACGAGAAGAGGCTTG
N_Sarb_P	FAM-ACTTCCTCAAGGAACAACATTGCCA-BHQ1
E_Sarb_F	ACAGGTACGTTAATAGTTAATAGCGT
E_Sarb_R	ATATTGCAGCAGTACGCACACA
E_Sarb_P	HEX-ACACTAGCCATCCTTACTGCGCTTCG-BHQ2
MS2-5-F	GTACGAGGAGAAAGCCGGTTTC
MS2-5-R	GTTCTGCGGCACTTCGATG
MS2-5-P	FAM-TCCCTCGACGCACGCTCCTGCT-BHQ1

**Table 2:** Time-to-threshold (Tt) values for LAMP with RNA template and primer sets CoV-2-E and MS2-2.

Concentration of Standard, Copies/Reaction	CoV-2-E	Concentration of Standard, Copies/Reaction	MS2-2
1 × 10^4^	7.88 ± 0.06	1250	13.18 ± 0.61
1 × 10^3^	8.68 ± 0.68	250	15.83 ± 0.12
50	10.37 ± 1.03	50	17.89 ± 0.34
5	12.56 ± 1.22	5	20.69 ± 2.28

**Table 3:** A comparison of results between multiplex MS2-CoV-2-E LAMP and real-time RT-PCR assays.

RT-PCR	Multiplex LAMP		Total
	Positive	Negative	
Positive	40	2	42
Negative	1	82	83
Total	41	84	125

## Data Availability

The data presented in this study are available on request from the corresponding author. The data are not publicly available due to ethical restrictions.

## Supplementary Materials

The following are available online at https://www.mdpi.com/article/10.3390/ijms22115743/s1, Figure S1: LAMP with MS2 monoplex, duplex and various MS2 RNA concentrations, Supplementary file.

## References

[1] Fire A., Xu S.Q. (1995). Rolling replication of short DNA circles. Proc. Natl. Acad. Sci. USA.

[2] Compton J. (1991). Nucleic acid sequence-based amplification. Nature.

[3] Piepenburg O., Williams C.H., Stemple D.L., Armes N.A. (2006). DNA detection using recombination proteins. PLoS Biol..

[4] Vincent M., Xu Y., Kong H. (2004). Helicase-dependent isothermal DNA amplification. EMBO Rep..

[5] Walker G.T., Fraiser M.S., Schram J.L., Little M.C., Nadeau J.G., Malinowski D.P. (1992). Strand displacement amplification—An isothermal, in vitro DNA amplification technique. Nucleic Acids Res..

[6] Dean F.B., Hosono S., Fang L., Wu X., Faruqi A.-F., Bray-Ward P., Sun Z., Zong Q., Du Y., Du J. (2002). Comprehensive human genome amplification using multiple displacement amplification. Proc. Natl. Acad. Sci. USA.

[7] Liu W., Dong D., Yang Z., Zou D., Chen Z., Yuan J., Huang L. (2015). Polymerase Spiral Reaction (PSR): A novel isothermal nucleic acid amplification method. Sci. Rep..

[8] Notomi T., Okayama H., Masubuchi H., Yonekawa T., Watanabe K., Amino N., Hase T. (2000). Loop-mediated isothermal amplification of DNA. Nucleic Acids Res..

[9] Goto M., Honda E., Ogura A., Nomoto A., Hanaki K.-I. (2009). Colorimetric detection of loop-mediated isothermal amplification reaction by using hydroxy naphthol blue. Biotechniques.

[10] Tomita N., Mori Y., Kanda H., Notomi T. (2008). Loop-mediated isothermal amplification (LAMP) of gene sequences and simple visual detection of products. Nat. Protoc..

[11] Kouguchi Y., Fujiwara T., Teramoto M., Kuramoto M. (2010). Homogenous, real-time duplex loop-mediated isothermal amplification using a single fluorophore-labeled primer and an intercalator dye: Its application to the simultaneous detection of Shiga toxin genes 1 and 2 in Shiga toxigenic Escherichia coli isolates. Mol. Cell. Probes.

[12] Liu W., Huang S., Liu N., Dong D., Yang Z., Tang Y., Ma W., He X., Ao D., Xu Y. (2017). Establishment of an accurate and fast detection method using molecular beacons in loop-mediated isothermal amplification assay. Sci. Rep..

[13] Mori Y., Nagamine K., Tomita N., Notomi T. (2001). Detection of loop-mediated isothermal amplification reaction by turbidity derived from magnesium pyrophosphate formation. Biochem. Biophys. Res. Commun..

[14] Veigas B., Branquinho R., Pinto J., Wojcik P.J., Martins R., Fortunato E., Baptista P.V. (2014). Ion sensing (EIS) real-time quantitative monitorization of isothermal DNA amplification. Biosens. Bioelectron..

[15] Yongkiettrakul S., Kampeera J., Chareanchim W., Rattanajak R., Pornthanakasem W., Kiatpathomchai W., Kongkasuriyachai D. (2017). Simple detection of single nucleotide polymorphism in Plasmodium falciparum by SNP-LAMP assay combined with lateral flow dipstick. Parasitol. Int..

[16] World Health Organization (2016). The Use of Loop-Mediated Isothermal Amplification (TB-LAMP) for the Diagnosis of Pulmonary Tuberculosis: Policy Guidance.

[17] Guo X.G., Zhou Y.Z., Li Q., Wang W., Wen J.Z., Zheng L., Wang Q. (2018). Rapid and reliable diagnostic method to detect Zika virus by real-time fluorescence reverse transcription loop-mediated isothermal amplification. AMB Express.

[18] Poon L.L.M., Leung C.S.W., Chan K.H., Lee J.H.C., Yuen K.Y., Guan Y., Peiris J.S.M. (2005). Detection of human influenza A viruses by loop-mediated isothermal amplification. J. Clin. Microbiol..

[19] Broughton J., Deng X., Yu G., Fasching C., Singh J., Streithorst J., Granados A., Sotomayor-Gonzalez A., Zorn K., Gopez A. (2020). Rapid Detection of 2019 Novel Coronavirus SARS-CoV-2 Using a CRISPR-based DETECTR Lateral Flow Assay. MedRxiv.

[20] Park G.-S., Ku K., Baek S.-H., Kim S.-J., Kim S.I., Kim B.-T., Maeng J.-S. (2020). Development of Reverse Transcription Loop-mediated Isothermal Amplification (RT-LAMP) Assays Targeting SARS-CoV-2. J. Mol. Diagn..

[21] Kitagawa Y., Orihara Y., Kawamura R., Imai K., Sakai J., Tarumoto N., Matsuoka M., Takeuchi S., Maesaki S., Maeda T. (2020). Evaluation of rapid diagnosis of novel coronavirus disease (COVID-19) using loop-mediated isothermal amplification. J. Clin. Virol..

[22] Schermer B., Fabretti F., Damagnez M., Di Cristanziano V., Heger E., Arjune S., Tanner N.A., Imhof T., Koch M., Ladha A. (2020). Rapid SARS-CoV-2 testing in primary material based on a novel multiplex RT-LAMP assay. PLoS ONE.

[23] Lalli M.A., Langmade J.S., Chen X., Fronick C.C., Sawyer C.S., Burcea L.C., Wilkinson M.N., Fulton R.S., Heinz M., Buchser W.J. (2021). Rapid and extraction-free detection of SARS-CoV-2 from saliva by colorimetric reverse-transcription loop-mediated isothermal amplification. Clin. Chem..

[24] Bektaş A., Covington M.F., Aidelberg G., Arce A., Matute T., Núñez I., Walsh J., Boutboul D., Delaugerre C., Lindner A.B. (2021). Accessible LAMP-enabled rapid test (ALERT) for detecting SARS-CoV-2. Viruses.

[25] Aliotta J.M., Pelletier J.J., Ware J.L., Moran L.S., Benner J.S., Kong H. (1996). Thermostable Bst DNA polymerase I lacks a 3′-->5′ proofreading exonuclease activity. Genet. Anal..

[26] Shao Y., Zhu S., Jin C., Chen F. (2011). Development of multiplex loop-mediated isothermal amplification-RFLP (mLAMP-RFLP) to detect Salmonella spp. and Shigella spp. in milk. Int. J. Food Microbiol..

[27] Iseki H., Alhassan A., Ohta N., Thekisoe O.M.M., Yokoyama N., Inoue N., Nambota A., Yasuda J., Igarashi I. (2007). Development of a multiplex loop-mediated isothermal amplification (mLAMP) method for the simultaneous detection of bovine Babesia parasites. J. Microbiol. Methods.

[28] Aonuma H., Yoshimura A., Kobayashi T., Okado K., Badolo A., Nelson B., Kanuka H., Fukumoto S. (2010). A single fluorescence-based LAMP reaction for identifying multiple parasites in mosquitoes. Exp. Parasitol..

[29] Higgins O., Clancy E., Cormican M., Boo T.W., Cunney R., Smith T.J. (2018). Evaluation of an internally controlled multiplex Tth endonuclease cleavage loop-mediated isothermal amplification (TEC-LAMP) assay for the detection of bacterial meningitis pathogens. Int. J. Mol. Sci..

[30] Yaren O., Alto B.W., Bradley K.M., Moussatche P., Glushakova L., Benner S.A. (2018). Multiplexed isothermal amplification based diagnostic platform to detect zika, chikungunya, and dengue 1. J. Vis. Exp..

[31] Dong J., Xu Q., Li C.C., Zhang C.Y. (2019). Single-color multiplexing by the integration of high-resolution melting pattern recognition with loop-mediated isothermal amplification. Chem. Commun..

[32] Tanner N.A., Zhang Y., Evans T.C. (2012). Simultaneous multiple target detection in real-time loop-mediated isothermal amplification. Biotechniques.

[33] Jang W.S., Lim D.H., Yoon J., Kim A., Lim M., Nam J., Yanagihara R., Ryu S.-W., Jung B.K., Ryoo N.-H. (2021). Development of a multiplex Loop-Mediated Isothermal Amplification (LAMP) assay for on-site diagnosis of SARS CoV-2. PLoS ONE.

[34] Zhang Y., Tanner N.A. (2021). Development of multiplexed reverse-transcription loop-mediated isothermal amplification for detection of SARS-CoV-2 and influenza viral RNA. Biotechniques.

[35] Corman V.M., Landt O., Kaiser M., Molenkamp R., Meijer A., Chu D.K., Bleicker T., Brünink S., Schneider J., Schmidt M.L. (2020). Detection of 2019 novel coronavirus (2019-nCoV) by real-time RT-PCR. Eurosurveillance.

[36] Hellou M.M., Górska A., Mazzaferri F., Cremonini E., Gentilotti E., De Nardo P., Poran I., Leeflang M., Tacconelli E., Paul M. (2020). Nucleic-acid-amplification tests from respiratory samples for the diagnosis of coronavirus infections: Systematic review and meta-analysis. Clin. Microbiol. Infect..

[37] Augustine R., Hasan A., Das S., Ahmed R., Mori Y., Notomi T., Kevadiya B., Thakor A. (2020). Loop-mediated isothermal amplification (LAMP): A rapid, sensitive, specific, and cost-effective point-of-care test for coronaviruses in the context of COVID-19 pandemic. Biology.

[38] Ahmad S., Ali N., Kausar M., Misbah H., Wahid A. (2020). Road toward rapid-molecular point of care test to detect novel SARS-coronavirus 2019 (COVID-19): Review from updated literature. Allergol. Immunopathol..

[39] Xu G., Gunson R.N., Cooper J.M., Reboud J. (2015). Rapid ultrasonic isothermal amplification of DNA with multiplexed melting analysis-applications in the clinical diagnosis of sexually transmitted diseases. Chem. Commun..

[40] Markoulatos P., Siafakas N., Moncany M. (2002). Multiplex polymerase chain reaction: A practical approach. J. Clin. Lab. Anal..

[41] Elnifro E.M., Ashshi A.M., Cooper R.J., Klapper P.E. (2000). Multiplex PCR: Optimization and application in diagnostic virology. Clin. Microbiol. Rev..

