# Supplementary material for: Detection of SARS-CoV-2 RNA by a Multiplex Reverse-Transcription Loop-Mediated Isothermal Amplification Coupled with Melting Curves Analysis
Source: Int J Mol Sci. 2021 May 27;22(11):5743. doi: 10.3390/ijms22115743 (PMC8197939; doi:10.3390/ijms22115743)
Supplement: Supplementary file 1 [file ijms-22-05743-s001.zip › Supplementary.pdf]

Standard samples

Standard plasmids with genome fragments of SARS-CoV-2 and MS2 phage

The 200 nt DNA fragments of SARS-CoV-2 E- and SARS-CoV-2 N-genes complementary to the SARS-CoV-2 RNA (GenBank ID NC_045512.2) were synthesized by PCR from separate oligonucleotides using the Polymerase Cycling Assembly (PCA) method (33). Oligonucleotides for assembly of CoV-2-E and CoV-2-N viral DNA fragments were synthesized on an automatic DNA/RNA synthesizer ASM-2000 (Biosset, Novosibirsk, Russia). The oligonucleotides for the assembly of extended DNA fragments were chosen by the GeneCut algorithm (ICBFM SB RAS – Unipro, Novosibirsk, Russia).

The obtained DNA fragments were hydrolyzed by restriction endonucleases EcoRI and HindIII (Sibenzyme, Novosibirsk, Russia) and ligated with the pBlueScript II SK (+) vector hydrolyzed by the same endonucleases, using 100 units of T4 DNA ligase (Biosan, Novosibirsk, Russia). Ligase mixture was then used to transform competent *E. coli* cells of XL1-Blue strain (Stratagene, CA, USA). The structure of plasmid clones was proved by a Sanger sequencing. The sequencing was performed on an ABI 3130XL GeneticAnalyzer (Applied Biosystems, MA, USA), using BigDye 3.1 kit (Genomics Core Facility, ICBFM SB RAS, Novosibirsk, Russia). The pBlueScript II SK (+) containing MS2 phage genome was synthesized artificially (Shanghai RealGene Bio-tech, Shanghai, China). Recombinant plasmid DNA pBlueScript-CoV-2-E, pBlueScript-CoV-2-N, and pBlueScript-MS2 were isolated from 50 mL of night cultures in LB medium with the use of QIAGEN Plasmid Midi Kit (QIAGEN, Venlo, Netherlands) according to the manufacturer’s protocol.

The concentrations of obtained standard plasmid DNAs were measured by s using the Qubit™ BR kit (Invitrogen, MA, USA). Then, 2 µg of each plasmid DNA were linearized by HindIII restriction endonuclease. The resulting linearized standards were diluted to the

concentration of 10^5 and further down to 2 copies of plasmid DNA per µL in a sterile buffer containing 10 mM Tris-HCl (pH 7.6) and 5 ng/mL yeast RNA.

In vitro RNA synthesis

Linearized using Hind III, pBlueScript-CoV-2-E and pBlueScript-CoV-2-N were served as templates for *in vitro* RNA synthesis. The reactions were carried out in a total reaction volume of 50 µL, containing 2 µg of DNA template, 1 mM of each NTP, 100 units of T7 RNA polymerase (Sibenzyme, Novosibirsk, Russia), 1x reaction buffer (50 mM Tris-HCl, pH 7,5, 6 mM $MgCl_2$, 10 mM DTT, 2 mM spermidine). After 2 h of incubation at 37 °C, 100 units of DNase I (Worthington Biochemical, NJ, USA) were added to the reaction mixtures and incubated for 15 min at 37 °C. Synthesized RNA fragments were isolated by phenol-chloroform extraction and precipitated with isopropanol. Purified RNAs were dissolved in DEPC-treated water and stored at -80 °C.

Isolation of MS2 phage RNA

The MS2 phage was grown using the modified protocol of Sambrook and Russel (34). The fresh night culture of *E. coli* K12 strain was diluted in 3 mL of MS2 medium to the OD_{600} = 1 (1×10^9 cells/mL), then MS2 phage was added to reach the phage/cell ratio of 5. The cultures were incubated at 37 °C for 20 min, mixed with 500 mL of pre-heated MS2 medium, and incubated under the same conditions for 12 h. The chloroform was added to lyse the cells, and the culture was vortexed for 10 min at 37 °C. The lysate was treated by DNase I and RNase A (50 mg/mL each) for 30 min at 37 °C, then NaCl was added to the final concentration of 1 M. The mixture was incubated on ice for 1 h, then the debris was separated by the centrifugation (10 min, 11000×*g*) at 4 °C. The supernatant was supplied by an additional amount of ammonium sulfate to the final concentration of 50% (m/m), the mixture was incubated for 2 h at 4 °C, then the phage particles were precipitated by

centrifugation (30 min, 11000×*g*) at 4 °C. The precipitated phage particles were dissolved in 30 mL of the TSM buffer. MS2 RNA was isolated from phage particles using QIAamp Circulating Nucleic Acid Kit (Qiagen, Germany) according to the manufacturer’s protocol and stored at -80 °C.

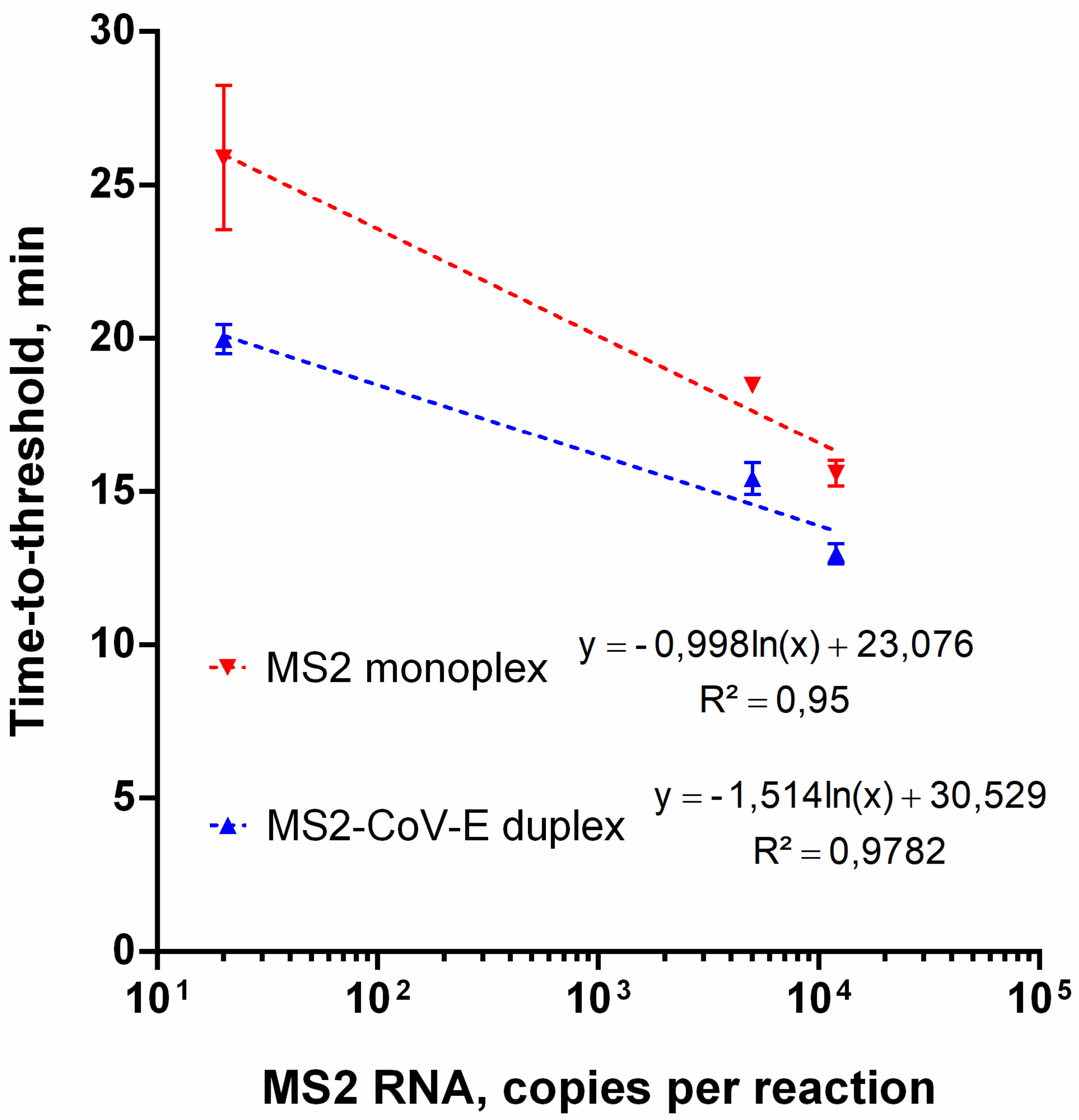

Figure S1

LAMP with MS2 monoplex, duplex and various MS2 RNA concentrations. Each MS2 RNA concentration is marked by the color, specified in the legend. Tt values are presented in X-axis, amount of MS2 RNA per reaction – in Y-axis. Each run was triplicated; error bars represent one SD.
